# Supplementary material for: What Pertussis Mortality Rates Make Maternal Acellular Pertussis Immunization Cost-Effective in Low- and Middle-Income Countries? A Decision Analysis
Source: Clin Infect Dis. 2016 Nov 2;63(Suppl 4):S227–35. doi: 10.1093/cid/ciw558 (PMC5106625; doi:10.1093/cid/ciw558)
Supplement: Supplementary Data [file supp_63_suppl-4_S227__index.html]

Supplementary Data 

# What Pertussis Mortality Rates Make Maternal Acellular Pertussis Immunization Cost-Effective in Low- and Middle-Income Countries? A Decision Analysis

## Supplementary Data

Supplementary Data

- Supplementary Data - Doc file
