# Supplementary material for: What Pertussis Mortality Rates Make Maternal Acellular Pertussis Immunization Cost-Effective in Low- and Middle-Income Countries? A Decision Analysis
Source: Clin Infect Dis. 2016 Nov 2;63(Suppl 4):S227–35. doi: 10.1093/cid/ciw558 (PMC5106625; doi:10.1093/cid/ciw558)
Supplement: Supplementary Data [file supp_ciw558_ciw558supp.doc]

**Table of Contents**

Appendix 1: Overview and model schematic

Appendix 2: Tables of model parameter values and sources

Table A2.1 Vaccination probability and efficacy parameters, by country

Table A2.2 Mortality parameters, by country

Table A2.3 Cost parameters, by country

Appendix 3: Vaccination probabilities

Appendix 4: Vaccine efficacy

Appendix 5: Pertussis mortality and death from other causes

Appendix 6: Costs: cost of infant vaccine; cost of delivery of infant vaccine; cost of maternal vaccine; cost of delivery of maternal vaccine; cost of treating hospitalized severe/fatal pertussis cases.

References

Appendix 1: Overview

The model, which covers the first year of infant life, focuses on the value for reducing deaths in infants of maternal aP immunization as a supplement to routine infant vaccination.

The model is shown in Figure A1.1. At the far left, the root node is labeled “Maternal Immunization?” The two branches that follow represent the two strategies modeled in the paper: Maternal immunization plus routine infant vaccination; and Routine infant vaccination alone. The upper branch, maternal immunization plus routine infant vaccination, first divides according to whether the mother receives aP immunization or not (maternal aP coverage). After that point the two strategies are identical: both model the probability that the infant receives routine DTP (diphtheria-tetanus-pertussis) vaccination in a given time interval and the health outcomes that occur in that time interval. The branches that follow “Maternal immunization not received” and “Routine infant vaccination alone” are the same as the branches that follow “Healthy 0-1 month” after “Maternal immunization received”, and thus are not shown. The only difference is that in the branch “Maternal immunization not received” a higher probability is assigned to the probability of dying of pertussis during the first three months of life because the mother was not vaccinated.

The model incorporates the following features.

1. The time intervals modeled are 0-1; 2-3; 4-5; 6-8; and 9-11 months of infant age.
2. The first step modeled in each time interval is the probability that the infant receives protection against pertussis, either through maternal immunization or through routine infant vaccination.
3. Following receipt (or not) of protection against pertussis, the infant may experience one of three outcomes: death from pertussis; death from other causes; or good health. Infants who contract pertussis or other diseases, but do not die are counted as experiencing good health.
4. If the infant is in good health, the same choices repeat at the next time interval, starting with whether the infant receives DTP vaccine during the time interval.
5. Vaccination is modeled by dose so that an infant who does not receive a scheduled dose in one time interval is eligible to receive it at the next time interval.

The choice between maternal immunization plus routine infant vaccination and routine infant vaccination alone is modeled for three countries: Bangladesh, Nigeria, and Brazil. Tables A2.1, A2.2, and A2.3 in Appendix 2 show the parameter estimates used for each country.

Figure A1.1: Model schematic (*Pertussis simple model mortality FINAL 16May2016.trex*)
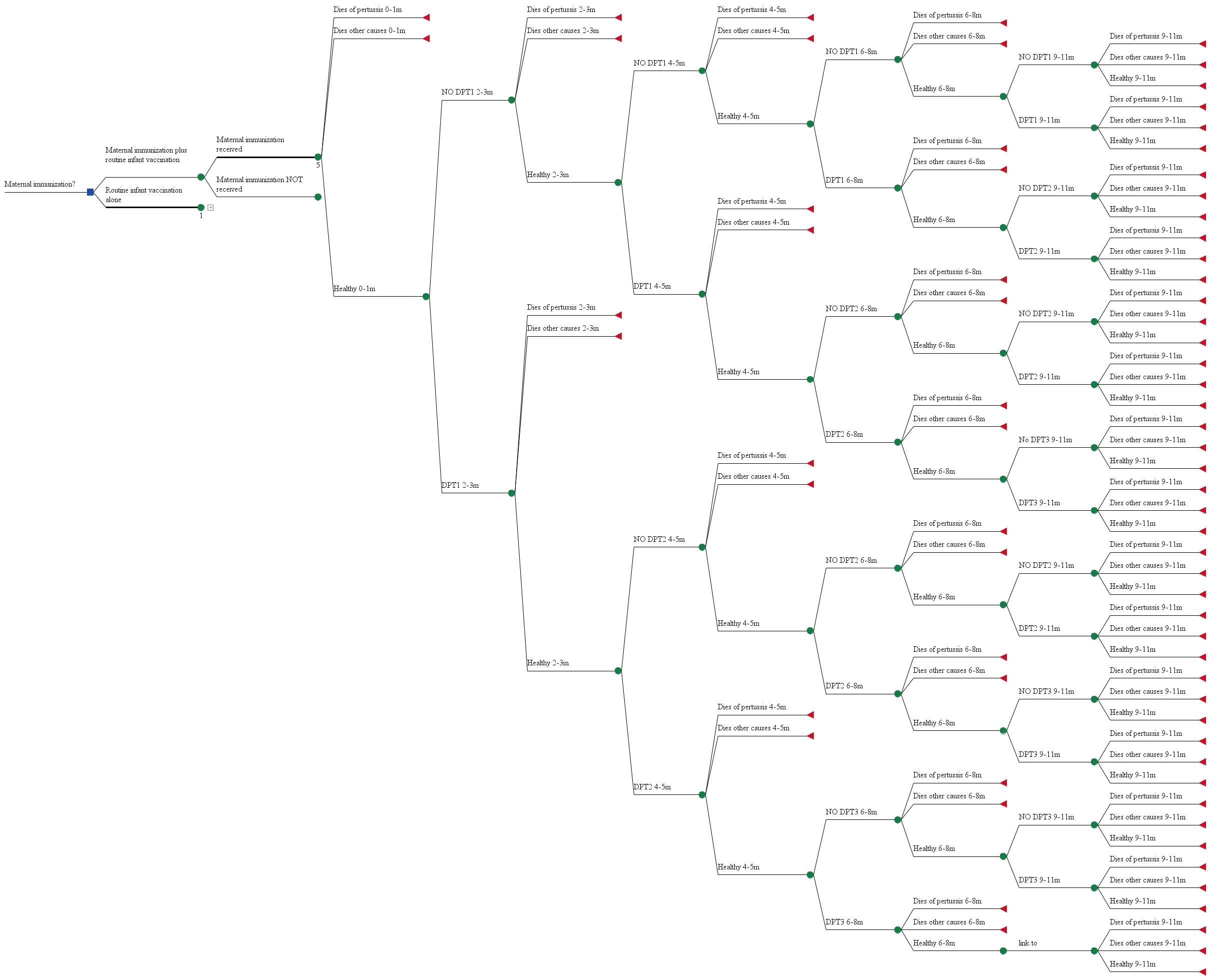


Appendix 2: Tables of model parameter values and sources.

Model parameters are presented in three tables: vaccination probabilities and efficacy (Table A2.1), pertussis and non-pertussis mortality (Table A2.2), and immunization and disease costs (Table A2.3). Each table shows the base-case value for each parameter, by country, the standard error or range considered, the type of distribution assigned to that parameter in the probabilistic model, and the data source.

Table A2.1. Vaccination probability and efficacy parameters, by country. NA = Not Applicable.

| **Name of parameter** | **Description of parameter** | **Bangladesh** |  | **Nigeria** |  | **Brazil** |  | **Distribution** | **Source** |
| --- | --- | --- | --- | --- | --- | --- | --- | --- | --- |
|  |  | **base case** | **SE/range** | **base case** | **SE/range** | **base case** | **SE/range** | Blank=fixed or none needed |  |
|  |  |  |  |  |  |  |  |  |  |
| p_dtp1 | Probability of receiving DTP1  **Always replaced** with probability for the age group |  |  |  |  |  |  |  |  |
| p_dtp1_23m | probability of receiving DTP1 at 2-3m | 0.810 | 0.0097 | 0.340 | 0.0062 | 0.940 | 0.0018 | beta | 1-6 |
| p_dtp1_45m | probability of receiving DTP1 at 4-5m | 0.737 | 0.0109 | 0.106 | 0.0040 | 0.360 | 0.0037 | beta | 1-6 |
| p_dtp1_68m | probability of receiving DTP1 at 6-8m | 0.400 | 0.0121 | 0.068 | 0.0033 | 0.160 | 0.0028 | beta | 1-6 |
| p_dtp1_911m | probability of receiving DTP1 at 9-11m | 0.200 | 0.0099 | 0.055 | 0.0030 | 0.040 | 0.0015 | beta | 1-6 |
| p_dtp2 | Probability of receiving DTP2  **Always replaced** with probability for the age group |  |  |  |  |  |  |  |  |
| p_dtp2_45m | probability of receiving DTP2 at 4-5m if DTP1 received | 0.925 | 0.0065 | 0.821 | 0.0050 | 0.920 | 0.0021 | beta | 1-6 |
| p_dtp2_68m | probability of receiving DTP2 at 6-8m if DTP1 received | 0.636 | 0.0119 | 0.417 | 0.0064 | 0.790 | 0.0031 | beta | 1-6 |
| p_dtp2_911m | probability of receiving DTP2 at 9-11m if DTP1 received | 0.200 | 0.0099 | 0.300 | 0.0060 | 0.410 | 0.0037 | beta | 1-6 |
| p_dtp3 | Probability of receiving DTP3  **Always replaced** with probability for the age group |  |  |  |  |  |  |  |  |
| p_dtp3_68m | probability of receiving DTP3 at 6-8m if DTP2 received | 0.924 | 0.0066 | 0.750 | 0.0056 | 0.940 | 0.0018 | beta | 1-6 |
| p_dtp3_911m | probability of receiving DTP3 at 9-11m if DTP2 received | 0.667 | 0.0117 | 0.417 | 0.0064 | 0.650 | 0.0036 | beta | 1-6 |
| p_matern_vax | probability mother/pregnant woman receives aP vaccine: ANC1 for Bangladesh and Nigeria, actual coverage for Brazil | 0.786 | 0.0059 | 0.658 | 0.0033 | 0.530 | 0.0287 | beta | 5,6 |
|  | probability mother/pregnant woman receives aP vaccine: TT2 (Td/TdaP for Brazil) | 0.980 | 0.0020 | 0.440 | 0.0035 | 0.530 | 0.0287 | beta | 7,8 |
| p_uncond_dtp1_23m | unconditional probability of only DTP1 at 2-3m | 0.536 | 0.0123 | 0.187 | 0.0051 | 0.937 | 0.0019 | dirichlet23 | 1-6 |
| p_uncond_dtp1_45m | unconditional probability of only DTP1 at 4-5m | 0.090 | 0.0071 | 0.090 | 0.0037 | 0.097 | 0.0023 | dirichlet45 | 1-6 |
| p_uncond_dtp1_68m | unconditional probability of only DTP1 at 6-8m | 0.040 | 0.0048 | 0.080 | 0.0035 | 0.006 | 0.0006 | dirichlet68 | 1-6 |
| p_uncond_dtp1_911m | unconditional probability of only DTP1 at 9-11m | 0.030 | 0.0042 | 0.080 | 0.0035 | 0.002 | 0.0003 | dirichlet911 | 1-6 |
| p_uncond_dtp23_23m | unconditional probability of DTP2/3 at 2-3m | 0.274 | 0.0110 | 0.153 | 0.0047 | 0.000 | 0.0000 | dirichlet23 | 1-6 |
| p_uncond_dtp23_45m | unconditional probability of DTP2/3 at 4-5m | 0.860 | 0.0086 | 0.320 | 0.0061 | 0.862 | 0.0026 | dirichlet45 | 1-6 |
| p_uncond_dtp23_68m | unconditional probability of DTP2/3 at 6-8m | 0.930 | 0.0063 | 0.370 | 0.0063 | 0.939 | 0.0018 | dirichlet68 | 1-6 |
| p_uncond_dtp23_911m | unconditional probability of DTP2/3 at 9-11m | 0.940 | 0.0059 | 0.400 | 0.0064 | 0.950 | 0.0017 | dirichlet911 | 1-6 |
| p_uncond_nodtp_23m | unconditional probability of no infant DTP at 2-3m | Derived: 1 - p_uncond_dtp1_23m - p_uncond_dtp23_23m | | | | | | dirichlet23 | NA |
| p_uncond_nodtp_45m | unconditional probability of no infant DTP at 4-5m | Derived: 1 - p_uncond_dtp1_45m - p_uncond_dtp23_45m | | | | | | dirichlet45 | NA |
| p_uncond_nodtp_68m | unconditional probability of no infant DTP at 6-8m | Derived: 1 - p_uncond_dtp1_68m - p_uncond_dtp23_68m | | | | | | dirichlet68 | NA |
| p_uncond_nodtp_911m | unconditional probability of no infant DTP at 9-11m | Derived: 1 - p_uncond_dtp1_911m - p_uncond_dtp23_911m | | | | | | dirichlet911 | NA |
| RR_matern | relative risk of child pertussis death if mother received aP vaccine | 0.15 | 0.03 | 0.15 | 0.03 | 0.15 | 0.03 | beta | 9 |
| RRdtp1 | relative risk of child pertussis death if infant received DTP1 | 0.32 | 0.09 | 0.32 | 0.09 | 0.32 | 0.09 | beta | 10 |
| RRdtp23 | relative risk of child pertussis death if infant received DTP2/3 | 0.05 | 0.02 | 0.05 | 0.02 | 0.05 | 0.02 | beta | 10 |
| N_infantvax_doses | Number of infant doses of DTP received | Set at 0, 1, 2, or 3 at terminal nodes and multiplied by c_infant_vax_total to yield total vaccination cost | | | | | |  | NA |
| N_maternvax | Number of maternal doses of aP or TdaP received. | Set at 0 or 1 at terminal nodes, as appropriate for the model arm | | | | | |  | NA |

Table A2.2. Pertussis and non-pertussis mortality parameters, by country. NA = Not Applicable

| **Name of parameter** | **Description of parameter** | **Bangladesh** |  | **Nigeria** |  | **Brazil** |  | **Distribution** | **Source** |
| --- | --- | --- | --- | --- | --- | --- | --- | --- | --- |
|  |  | **base case** | **SE/range** | **base case** | **SE/range** | **base case** | **SE/range** | Blank=fixed or none needed |  |
| LE | Life expectancy at birth | 71.01 | 69.04-72.88 | 52.29 | 50.18-53.73 | 74.05 | 72.58-75.42 | uniform | 11 |
| LE_discounted | Life expectancy at birth, discounted at 3% | 27.82 | 27.25-28.25 | 22.94 | 22.21-23.43 | 28.47 | 28.20-28.74 | uniform | 12 |
|  | Life expectancy at birth, discounted at 5% | 18.39 | 18.05-18.62 | 15.78 | 15.33-16.08 | 18.75 | 18.62-18.88 | uniform | 12 |
| MR1 | probability of dying from pertussis in the first year | Ranged to identify cost-effectiveness thresholds | | | | | | uniform | NA |
| p_die_other | Probability of dying from other causes  **Always replaced** with probability for the age group |  |  |  |  |  |  |  |  |
| p_die_other_01m | probability of dying of other causes 0-1m | 0.02492 |  | 0.03838 |  | 0.01004 |  |  | 13 |
| p_die_other_23m | probability of dying of other causes 2-3m | 0.00148 |  | 0.00700 |  | 0.00088 |  |  | 13 |
| p_die_other_45m | probability of dying of other causes 4-5m | 0.00148 |  | 0.00700 |  | 0.00088 |  |  | 13 |
| p_die_other_68m | probability of dying of other causes 6-8m | 0.00222 |  | 0.01048 |  | 0.00132 |  |  | 13 |
| p_die_other_911m | probability of dying of other causes 9-11m | 0.00222 |  | 0.01048 |  | 0.00132 |  |  | 13 |
| p_perts_mort | Probability of dying of pertussis  **Always replaced** with probability for the age group |  | | | | | |  |  |
| p_pertsmort_01m | probability infant 0-1m dies of pertussis | Derived: MR1*0.625, where MR1=probability of dying of pertussis in the first year | | | | | |  | 14 |
| p_pertsmort_23m | probability infant 2-3m dies of pertussis | Derived: MR1*0.291667 | | | | | |  | 14 |
| p_pertsmort_45m | probability infant 4-5m dies of pertussis | Derived: MR1*0.0625 | | | | | |  | 14 |
| p_pertsmort_68m | probability infant 6-8m dies of pertussis | Derived: MR1*0.010417 | | | | | |  | 14 |
| p_pertsmort_911m | probability infant 9-11m dies of pertussis | Derived: MR1*0.010417 | | | | | |  | 14 |

| **Name of parameter** | **Description of parameter** | **Bangladesh** |  | **Nigeria** |  | **Brazil** |  | **Distribution** | **Source** |
| --- | --- | --- | --- | --- | --- | --- | --- | --- | --- |
|  |  | **base case** | **SE/range** | **base case** | **SE/range** | **base case** | **SE/range** | Blank=fixed or none needed |  |
| p_pertsmort_dtp1_23m | probability of dying of pertussis at 2-3m if received DTP1 | Derived: p_pertsmort_nodtp_23m*RRdtp1 | | | | | |  | NA |
| p_pertsmort_dtp1_45m | probability of dying of pertussis at 4-5m if received DTP1 | Derived: p_pertsmort_nodtp_45m*RRdtp1 | | | | | |  | NA |
| p_pertsmort_dtp1_68m | probability of dying of pertussis at 6-8m if received DTP1 | Derived: p_pertsmort_nodtp_68m*RRdtp1 | | | | | |  | NA |
| p_pertsmort_dtp1_911m | probability of dying of pertussis at 9-11m if received DTP1 | Derived: p_pertsmort_nodtp_911m*RRdtp1 | | | | | |  | NA |
| p_pertsmort_dtp23_23m | probability of dying of pertussis at 2-3m if received DTP2/3 | Derived: p_pertsmort_nodtp_23m*RRdtp23 | | | | | |  | NA |
| p_pertsmort_dtp23_45m | probability of dying of pertussis at 4-5m if received DTP2/3 | Derived: p_pertsmort_nodtp_45m*RRdtp23 | | | | | |  | NA |
| p_pertsmort_dtp23_68m | probability of dying of pertussis at 6-8m if received DTP2/3 | Derived: p_pertsmort_nodtp_68m*RRdtp23 | | | | | |  | NA |
| p_pertsmort_dtp23_911m | probability of dying pertussis at 9-11m if received DTP2/3 | Derived: p_pertsmort_nodtp_911m*RRdtp23 | | | | | |  | NA |
| p_pertsmort_nodtp_23m | probability of dying of pertussis at 2-3m if no DTP | Derived: p_pertsmort_23m/(p_uncond_nodtp_23m*RRnodtp + p_uncond_dtp1_23m*RRdtp1 + p_uncond_dtp23_23m*RRdtp23) | | | | | |  | NA |
| p_pertsmort_nodtp_45m | probability of dying of pertussis at 4-5m if no DTP | Derived: p_pertsmort_45m/(p_uncond_nodtp_45m*RRnodtp + p_uncond_dtp1_45m*RRdtp1 + p_uncond_dtp23_45m*RRdtp23) | | | | | |  | NA |
| p_pertsmort_nodtp_68m | probability of dying of pertussis at 6-8m if no DTP | Derived: p_pertsmort_68m/(p_uncond_nodtp_68m*RRnodtp + p_uncond_dtp1_68m*RRdtp1 + p_uncond_dtp23_68m*RRdtp23) | | | | | |  | NA |
| p_pertsmort_nodtp_911m | probability of dying of pertussis at 9-11m if no DTP | Derived: p_pertsmort_911m/(p_uncond_nodtp_911m*RRnodtp + p_uncond_dtp1_911m*RRdtp1 + p_uncond_dtp23_911m*RRdpt23) | | | | | |  | NA |
| p_health | probability of surviving | Derived: 1 - p_perts_mort - p_die_other | | | | | |  | NA |

Table A2.3. Immunization and disease costs in 2014 U.S. dollars, by country

| **Name of parameter** | **Description of parameter** | **Bangladesh** |  | **Nigeria** |  | **Brazil** |  | **Distribution** | **Source** |
| --- | --- | --- | --- | --- | --- | --- | --- | --- | --- |
|  |  | **base case** | **SE/range** | **base case** | **SE/range** | **base case** | **SE/range** | Blank=fixed or none needed |  |
| c_hosp | Hospital cost: WHO-CHOICE | 43.30 | 34.18-52.41 | 196.32 | 154.99-237.65 | 189.61 | 149.69-229.53 | uniform | 15,24,25 |
|  | Hospital cost: scaled to Brazil | 256.76 | 42.35 | 1164.25 | 192.03 | 1124.47 | 185.47 | normal | 14,15 |
| c_infant_vax_delivery | Delivery cost per dose for infant vaccination (+/-10%) | 0.74 | 0.67-0.81 | 5.83 | 5.25-6.41 | 5.97 | 5.37-6.57 | uniform | 16-18,  24,25 |
| c_infant_vax_price | UNICEF or PAHO price/dose for infant vaccine + insurance/freight + 5% wastage | 2.23 | 1.40-2.81 | 2.23 | 1.40-2.81 | 2.30 | 2.19-2.42 | uniform | 19-22 |
| c_infant_vax_total | Total cost of infant vaccination during the first year | Derived: N_infantvax_doses*(c_infant_vax_price + c_infant_vax_delivery) | | | | | |  | NA |
| c_matern_vax_delivery | Delivery cost per dose for maternal immunization | Assumed no incremental costs as maternal TdaP programs would replace current/proposed Td immunization programs; Rollout of Brazil’s TdaP program began in late 2014. | | | | | |  |  |
| c_matern_vax_price | Vaccine price/dose for maternal immunization | Set for each analysis as described in main paper. | | | | | | uniform |  |
| c_matern_vax_total | Total cost of maternal immunization | Derived: N_maternvax*(c_matern_vax_price + c_matern_vax_delivery) | | | | | |  | NA |
| c_vax_total | Total cost of maternal (if any) and infant vaccinations | Derived: c_matern_vax_total + c_infant_vax_total | | | | | |  | NA |

Appendix 3: Vaccination probabilities

Vaccine Coverage

Maternal aP coverage. To protect the infant maternal aP vaccine must be received late in the pregnancy for that infant. Thus, maternal aP vaccine would be offered late in pregnancy, either as part of antenatal care or through the same programs that currently provide women with tetanus toxoid (TT) or tetanus diphtheria (Td) vaccines.

In some LMICs many pregnant women first attend antenatal care (ANC) late in pregnancy and have only one visit before delivery. Thus ANC1, the percentage of pregnant women with at least one antenatal visit, is a reasonable proxy for vaccine coverage by this route. We used ANC1 from the most recent Demographic and Health Survey (DHS) as one measure of maternal vaccine coverage for Bangladesh (2014, 78.6%) and Nigeria (2013, 65.8%) [5,6]. We also considered an alternative proxy for maternal vaccine coverage for Bangladesh and Nigeria, the percentage of pregnant women who receive two or more doses of tetanus toxoid vaccine (TT2) [7]. It may, however, be less appropriate because tetanus toxoid protection can be based on vaccinations received before the current pregnancy. To obtain standard errors for these coverage estimates, we used the formula: SQRT(p*(1-p))/N, where p = proportion of women receiving ANC1/TT2; and N = base population of women for estimates of mothers protected against tetanus. The base population (weighted N) was 4,904 for Bangladesh in the 2014 DHS [5] and 20,467 for Nigeria in the 2013 DHS [6].

For Brazil, which began offering maternal aP immunization in late 2014, we use the national rate of coverage in 2015 (0.5303, or 53%) for either Td or TdaP [8] since, although the conversion is not yet complete, the goal is to replace Td with TdaP. We treated the tertiles of the coverage distribution among the 26 Brazilian states (48% and 59%) as the bounds of a 95% confidence interval around this mean in order to calculate the standard error of 0.0287.

Infant vaccine coverage: Bangladesh and Nigeria. Colin Sanderson, of the London School of Hygiene and Tropical Medicine/UK, generously provided us with data for a large number of LMICs showing the percentages of infants who received DTP1, DTP2, and DTP3, by week of age for the first three years of life in the early/mid-2000s [1]. The data are modeled from national Demographic and Health Surveys and are more recent than the percentages Clark and Sanderson recently reported [2]. We used these data to represent routine infant vaccination in Bangladesh and Nigeria, both of which use the 6, 10, 14 week vaccination schedule [23].

The weekly vaccination data were first categorized into the age intervals used in the model: 2-3 months (9-16 weeks), 4-5 months (17-26 weeks), 6-8 months (27-38 weeks), 9-11 months (39-52 weeks). Because vaccination in the model represents protection against pertussis we used the percentage vaccinated at the midpoint of each age interval, as this seems most likely to represent the proportion of children who have not only received the dose but developed immunity based on it: the mid-points for these intervals were 12, 21, 32, and 45 weeks, respectively. Probabilities of DTP1, DTP2, and DTP3 vaccination were calculated for each age interval in the model. For Bangladesh and Nigeria the numerator of each probability was children who received the specified dose in that age interval; the denominator was children in that age interval who did not receive the specified dose at an earlier age and thus were still eligible to receive it.

An example of the calculations for Bangladesh is provided, and the calculations explained, below.

Coverage data for Bangladesh, selected week

| Week | DTP1 | DTP2 | DTP3 |
| --- | --- | --- | --- |
| 0 | 0 | 0 | 0 |
| 9 | 0.56 | 0.0093 | 0.00069 |
| 12 | 0.81 | 0.27 | 0.0043 |
| 17 | 0.93 | 0.74 | 0.27 |
| 21 | 0.95 | 0.86 | 0.6 |
| 27 | 0.96 | 0.92 | 0.8 |
| 28 | 0.97 | 0.92 | 0.81 |
| 32 | 0.97 | 0.93 | 0.85 |
| 39 | 0.97 | 0.94 | 0.89 |
| 41 | 0.97 | 0.94 | 0.9 |
| 45 | 0.97 | 0.94 | 0.91 |
| 52 | 0.97 | 0.95 | 0.91 |

Calculation of DTP1 vaccination rates

- The probability of DTP1 vaccination (protection) for the 2-3 month age interval is simply 0.81, the proportion vaccinated by 12 weeks, the mid-point of the 2-3 month interval.
- The probability of DTP1 vaccination for the 4-5 month age interval, given that the infant had not received DTP1 earlier, was calculated by subtracting the proportion vaccinated at 12 weeks (0.81) from the proportion vaccinated at 21 weeks (0.95), to get the proportion vaccinated between 12 and 21 weeks, and dividing by those who had not received DTP1 by 12 weeks (1 – 0.81), yielding (0.95-0.81)/(1-0.81) = 0.737.
- The probabilities for vaccination with DTP1 in the 6-8 month and 9-11 month age intervals were calculated similarly, by calculating the number who received DTP1 between the midpoints of the two time intervals and dividing by the proportion who had not yet received DTP1.

Calculation of DTP2 and DTP3 vaccination rates

These vaccination rates recognized that, in Bangladesh (and Nigeria) an infant would not receive DTP2 until at least 4 weeks after receiving DTP1 and would not receive DTP3 until at least 4 weeks after receiving DTP2. As one example, for the probability of DTP2 vaccination in the 4-5 month age interval we used the DTP2 vaccination rate at 21 weeks and divided by the proportion of infants who received DTP1 at least 4 weeks earlier (at week 17), yielding (0.86/0.93)=0.925.

To obtain standard errors for these estimates, we used the formula: SQRT(p*(1-p))/N) where, p=proportion who received a given DTP dose in a specific age interval and N=base population of children aged 12-23 months, in DHS surveys, for estimates of the percentage who received 3 doses of DTP vaccine. The size of this population (weighted n) was 1,633 in the 2014 DHS for Bangladesh [5] and 5,900 in the 2013 DHS for Nigeria [6].

Infant vaccine coverage: Brazil. Vaccination rates for Brazil came from special analyses of 2014 data provided by the Brazilian Ministry of Health and Goiania Municipality Secretary of Health; the analyses were conducted by Cristiana Toscano, University of Goias/Brazil and her team [3, 4]. Brazil delivers DTP infant vaccine on a 2, 4, and 6 month schedule, the standard schedule in Latin America [23].

The calculations used data on vaccine doses delivered by age group in the municipality of Goiania [3], as such information is not available for the country as whole. Overall DTP coverage in Goiania is similar to national overall coverage. The numerator was the number of each specific dose delivered to children in the age interval. The denominator was the number of children in the age interval who had not yet received that dose, estimated from the national information system of live births and the data on doses received at earlier ages. In Brazil, which uses the 2/4/6 month schedule for DTP vaccination, the time between doses for calculating the probabilities of DTP2 and DTP3 was two months.

To obtain standard errors for these estimates, we used the formula: SQRT(p*(1-p))/N) where, p=proportion who received a given DTP dose in a specific age interval and N=17,214 infants from the municipality of Goiania [3].

Appendix 4: Vaccine Efficacy

Maternal aP vaccine efficacy. The estimate of maternal aP vaccination effectiveness comes from an English study [9], which found that maternal TdaP vaccine effectiveness in infants younger than 3 months was 91% (95% CI: 84 - 95). Because a single antigen (pertussis toxin) aP vaccine currently in development may be used in LMICs, we conservatively lowered this estimate to 85% for the current analyses and scaled the 95% CI proportionately.  We assume that maternal immunization protects infants for the first three months of life.

Infant vaccine efficacy. The efficacy of routine infant vaccine was taken from a study by Juretzko et al. [10] and is shown as proportions in the table below. We combined the data for the second and third doses and used an average efficacy of 0.95 (95%) for those doses in the model. We did not adjust the vaccine efficacy of DTP1 for any possible decrease or increase in protection from maternal immunization.

| **DTP** | **Vaccine efficacy (VE)** | **Relative risk (RR)** | **Standard deviation for VE and RR** | **Original source** |
| --- | --- | --- | --- | --- |
| **First dose** | 0.680 | 0.320 | 0.09 | (10) |
| **Second dose** | 0.918 | 0.082 | 0.03 |
| **Third dose** | 0.998 | 0.002 | 0.003 |

Appendix 5: Pertussis mortality and death from other causes

Health Outcomes

As noted, there are three possible outcomes during each age interval. The infant may die of pertussis, die of other causes, or be healthy and live an average life expectancy. Healthy is the residual after deaths from pertussis and other causes are deducted; it thus includes infants who have been ill with pertussis (or other diseases) but recovered. The probability of each outcome depends on the infant’s age and the number of doses of DTP the infant has received.

Death from pertussis. To serve the purpose of the analysis – to identify threshold rates of pertussis mortality that make maternal aP immunization cost-effective – death from pertussis is represented in three steps in the model. The first step allows the probability of death from pertussis during the first year to be set at any level, which makes it possible to explore any conceivable range of mortality rates in order to identify those at which maternal aP immunization is cost-effective by selected cost-effectiveness thresholds. The second and third steps, described in the following paragraphs, ensure that the overall pertussis mortality rate is appropriately distributed by age and vaccination status within the first year.

The age distribution of pertussis deaths in children under 1 year of age was obtained from Brazilian data on pertussis mortality in hospitalized children for the year 2014 [14] , shown in the table below. Pertussis deaths are concentrated in the youngest infants, with the majority occurring in the first month and over 90% in the first two months. The model thus calculates the pertussis death rate in an age interval as the product of the overall pertussis mortality rate for infants (which, as noted, can be set at any level) and the proportion of deaths that occurs in that age interval.

Age distribution of pertussis deaths within first year, %, 2014

Source: Brasil. DATASUS. Sistema de Informação de Hospitalizações (SIH-SUS)[14]

| Age categories | % Deaths |
| --- | --- |
| 0-1 months | 62.50 |
| 2-3 months | 29.17 |
| 4-5 months | 6.25 |
| 6-8 months | 1.04 |
| 9-11 months | 1.04 |

To account for vaccination status the third step applies equations that express the probability of death from pertussis in an age interval as a weighted average of the probabilities of death by vaccination status – no DTP, one dose of DTP, two or more doses of DTP. When populated with the unconditional probabilities of receiving each dose (Table A2.1), derived from the data supplied by Colin Sanderson [1], and vaccine efficacy [10] (also Table A2.1), the equations yield the probabilities of death by vaccination status within each age interval, thus completing the distribution of deaths by age and vaccination status.

The equations accomplish this result as follows. Equation 1 expresses the overall probability of dying of pertussis in age interval **i**, **p_perts_total_i**, as a weighted average of the probabilities for each vaccination status in that age interval: no vaccination, one dose, and 2 or more doses. In each of the three products on the right-hand side of the equation the first term is the probability of that vaccination status in that age interval and the second term is the probability of death for that vaccination status in that age interval.

**EQN 1: p_perts_total_i = p_dtp0_i * p_perts_i_nodtp + p_dtp1_i * p_perts_i_dtp1, + p_dtp2_i * p_perts_i_dtp2**

The first two steps described earlier provide an estimate of p_perts_total_i for the left-hand side of the equation. The (unconditional) probabilities of having received each dose were derived, as noted, from the data supplied by Colin Sanderson and are shown in Table A2.1.

That leaves three unknown terms, the probabilities of death for each vaccination status. Two more equations which express vaccine efficacy (VE) for each dose of DTP in terms of the relative risk of death yield a total of three equations, which can then be used to solve for the three unknowns. The two additional equations are

**EQN 2: VE_1 = 1 – (p_perts_i_dtp1/ p_perts_i_nodtp)**

**EQN 3: VE_2 = 1 – (p_perts_i_dtp2/ p_perts_i_nodtp)**

where “1” refers to DTP1 and “2” to DTP23. The ratio on the right-hand side of each equation is the relative risk of death for an infant who has received that number of doses of DTT, RR_1 and RR_2, respectively. Vaccine efficacies and relative risks are available from [10] and relative risks are shown in Table A2.1

With these three equations in hand return to Equation 1. First, multiply the entire right-hand side by p_perts_i_nodtp. Then divide the second term in each product inside the parentheses by the same term, p_perts_i_nodtp. Doing so does not change the equation, but allows it to be restated as

**EQN 1: p_perts_total_i = p_perts_i_nodtp [p_dtp0_i*1 + p_dtp1_i*RR_1 + p_dtp2_i*RR_2]**

As noted, values are available for all the variables in the restated equation:

- The left-hand side variable, overall probability of dying of pertussis in the age interval;
- The three (unconditional) probabilities that an infant has no, 1, or 2/3 dose of DTP;
- The two relative risks associated with DTP1 and DTP2/3.

Dividing both sides of Equation 1 by the sum in brackets yields an estimate of p_perts_i_nodtp.

**p_perts_i_nodtp = p_perts_total_i/ [p_dtp0_i * 1 + p_dtp1_i *RR_1 + p_dtp2_i * RR_2]**

When that estimate is inserted into Equations 2 and 3, they yield estimates of p_perts_i_dtp1 and p_perts_i_dtp2, completing the estimates of pertussis mortality by age and vaccination status.

Death from other causes. The probabilities of death from other causes for each age interval were calculated for all three countries using the United Nations Inter-Agency Group’s mortality estimates for neonates and infants ([www.childmortality.org](http://www.childmortality.org/)) [13]. After neonatal mortality (NMR) was subtracted from infant mortality (IMR) the remainder was distributed evenly over months 2 through 11.

More specifically, the mortality rate for the entire period 2-11 months, MR_211, was calculated as (IMR - NMR)/(1000 - NMR). The average *monthly* rate of death from other causes for the period 2-11 months, AMR_211, was then obtained using the formula –(LN(1-MR_211))/11. The probability of death from other causes in the age interval 0-1 month is neonatal mortality for the first month plus AMR_211 for the second month. Probabilities of death from other causes for the remaining age intervals were calculated using AMR_211 for the appropriate number of months, two months for the age intervals 2-3 and 4-5 months, three months for the age intervals 6-8 and 9-11 months.

Here is an example of the calculations for Bangladesh:

Step 1. For year 2014, NMR and IMR were 24.2 and 32.1 per 1000 live births, respectively. Thus at the end of one year 967.9 of 1000 infants born alive were still alive (1000 – 32.1). This number provides a check on the calculations for the age intervals since they should yield that many live infants at the end of the year.

Step 2. MR_211 = (32.1-24.2)/(1000-24.2)= 0.0081.

Step 3. AMR_211 = -(LN(1-0.0081))/11 = 0.000739.

Step 4. The NMR shows that 24.2 infants died in the first month after birth, leaving 975.80 live infants (1000-24.2) at the end of the first month. Of these 0.721 were estimated to die in the next month (975.80*0.000739), so, in all 24.921 infants died of other causes in the first two months of life, yielding a probability of death from other causes in that age interval of 0.02492 (24.921/1000) and leaving 975.08 alive at the end of the age interval. .

Step 5. For each month AMR_211 was applied to the number of infants alive at the beginning of the month to calculate the number who died during the month and the number alive at the beginning of the next month.

Step 6. Numbers of deaths for each month were aggregated into the age intervals used in the model and probabilities calculated by dividing the number of deaths by the number of infants alive at the beginning at the age interval.

| Month | Live Infants at end of month | Probability of death from other causes by age interval |
| --- | --- | --- |
| 0 | 975.80 | 0.02492 |
| 1 | 975.08 |
| 2 | 974.36 | 0.00148 |
| 3 | 973.64 |
| 4 | 972.92 | 0.00148 |
| 5 | 972.20 |
| 6 | 971.48 | 0.00222 |
| 7 | 970.76 |
| 8 | 970.05 |
| 9 | 969.33 | 0.00222 |
| 10 | 968.61 |
| 11 | 967.90 |

Healthy. Life expectancy at birth for healthy children was obtained from the United Nations’ Population Division, World Population Prospects: The 2015 Revision, File MORT/16-1: Life expectancy at exact age, e(x), for both sexes combined, by major area, region and country, 1950-2100 [11]. Our base-case estimates represent life-expectancy estimates for the period 2010-2015. Life expectancies at birth for the periods, 2005-2010 and 2015-2020 provide the lower and upper bounds for the base case.

Discounted life expectancy (again, 2010-2015 as base case) for use in deriving cost-effectiveness ratios was calculated using discount rates of 3% and 5% and the data on probabilities of survival to each year of age from the United Nations’ World Population Prospects: The 2015 Revision, File MORT/15-1: Life table survivors at exact age, l(x), for both sexes combined, by major area, region and country, 1950-2100 [12]. Numbers of survivors, beginning with 100,000, are available from this file for ages 0, 1, and 5, and at 5-year intervals thereafter up to 100 years of age; we derived values for the intervening years by linear interpolation. Survival probabilities were then calculated by dividing the number of survivors each year by 100,000. Summing those probabilities provides undiscounted life expectancy and served as check on the calculations. To discount life expectancy at 3% per year the survival probabilities were divided by 1.03 (1 plus the discount rate) raised to the power given by the year of age, and summed over all 100 years. Life expectancy discounted at 5% was calculated the same way, with 3% replaced by 5%.

Appendix 6: Costs

All costs are expressed in 2014 U.S. dollars. The World Bank’s Inflation Annual Deflator series [24] and Annual Average Currency Exchange [25] files were used to obtain data on inflation rates and currency exchange rates.

Price of infant vaccine, per dose. The price of infant vaccine, per dose, for Bangladesh and Nigeria was obtained from the 2016 price list of UNICEF Supply Division [19]. The average of all the per-dose prices listed for pentavalent DTP-HepB-Hib vaccine was used in the base-case analysis, with the lowest and highest listed prices serving as lower and upper bounds for the range. A mark-up of 17.9%, which included handling fees (1.4%), freight and insurance (5.5%), wastage (5%), and contingency Buffer (6%), was applied to arrive at the fully loaded vaccine price [20,21]. The final per-dose cost of infant vaccine for these two countries was $2.23 ($1.40-$2.81).

Brazil sources vaccines for its EPI program through the Pan American Health Organization Revolving Fund. We obtained the per-dose cost of infant DTP vaccine for Brazil from the 2015 PAHO Amendment-1 price list for EPI vaccines [22]. After a mark-up of 3% for insurance and freight and an additional 5% for wastage, the final cost estimate was $2.30 per dose ($2.19-$2.42). The upper and lower bounds for Brazil were estimated as +/-5% of $2.30.

Delivery cost of infant vaccine, per dose. The costs of vaccine delivery, per dose, were estimated for Bangladesh and Nigeria based on data obtained from the comprehensive Multi-Year Plans (cMYPs) for 2009 (Bangladesh) and 2005 (Nigeria) [16]. We used routine recurrent costs, routine capital costs, and number of vaccines delivered to arrive at the average delivery cost, per dose, within the EPI program. Baseline-year costs, reported in U.S. Dollars, were converted into local currency [25], adjusted for inflation [24], and back converted to 2014 U.S. Dollars [25]. Lower and upper bounds were calculated as -/+10% of the average. The final estimates were $0.74 for Bangladesh ($0.67-$0.81) and $5.83 for Nigeria ($5.25-$6.41).

There was no cMYP available for Brazil. We used $5.97 as the per-dose delivery cost for infant vaccine based on comprehensive costing studies of routine immunization programs in Honduras and Colombia [17, 18]. Again, -/+10% was used to estimate lower and upper bounds.

Price of maternal vaccine, per dose. A less costly formulation for maternal aP vaccine is under development, which will contain tetanus, diphtheria, and a single component of pertussis, pertussis toxin. As the vaccine is under development there is no market price available. The Gates Foundation was interested in understanding the impact on the cost-effectiveness of maternal aP immunization of a wide price range, starting as low as $.50 per dose. The prices used in our analysis include the standard 5% wastage rate recommended by WHO.

Brazil started its TdaP maternal vaccination program in late 2014 using a multivalent formulation purchased through the Pan American Health Organization Revolving Fund [22]. . The per-dose cost of maternal TdaP vaccine for Brazil was derived by applying 3% for insurance and freight and 5% for wastage mark-up to the price of the maternal TdaP in PAHO’s price list [22], resulting in a base-case cost of $11.50 per dose. The analyses reported in the paper also explore the effects of lower prices in the expectation that Brazilian policy makers might be interested in the lower-cost vaccine being developed by Gates/GAVI.

Delivery cost of maternal vaccine, per dose. Since Brazil is simply replacing Td with TdaP in its current maternal immunization program, incremental delivery costs for TdaP are expected to be zero. Along similar lines, according to their latest cMYPs [16], Bangladesh and Nigeria expect to roll out their own maternal Td immunization programs by replacing tetanus toxoid (TT) with Td. Thus, again, replacing Td with TdaP is not expected to involve additional delivery costs. We note that any minor incremental increase in maternal vaccine delivery costs is accommodated by the wide range of maternal vaccine costs used in our analyses, but coverage rates higher than those current in each country would likely involve additional system-level costs that would need to be accounted for.

Cost of treating hospitalized severe/fatal pertussis cases. Several sources were used to estimate the cost of treating hospitalized pertussis cases in Bangladesh and Nigeria. We first obtained bed-day cost at a secondary level hospital in 2008 U.S. Dollars from WHO CHOICE [15]. These cost estimates were converted to local currency in 2008 [25]; adjusted for inflation through 2008-14 [24]; and re-converted to 2014 U.S. Dollars [25].

Since the WHO CHOICE cost estimates represent only facility and personnel costs, we applied mark-ups for procedure costs (0.49%), diagnostic tests (16.77%), and treatment costs (5.14%) based on [26](. The sum of these 4 costs (facility and personnel, procedures, diagnostic tests, and treatment costs) gave us the total per-day cost of a hospitalized severe pertussis case. Based on our systematic literature review on infant pertussis mortality and morbidity in low- and middle- income countries, we used 6.0-9.2 days as range for length of stay for hospitalization due to severe pertussis [27]. Total treatment costs were estimated by multiplying per-day costs by length of stay, and the average of the range was used for base-case analyses. The final estimates for cost of treating hospitalized severe pertussis cases in 2014 U.S. Dollars were: Bangladesh ($34.18-$52.41), Nigeria ($154.99-$237.65), and Brazil ($149.69-$229.53).

For Brazil, we used 2014 data on reimbursements for inpatient treatment for children under 12 months hospitalized due to pertussis in public health system hospitals (which cover 75% of the Brazilian population) who died of pertussis [14]. The average reported inpatient treatment cost for fatal pertussis was $1,124, which is much higher than the $190 ($150 to $230) estimated for Brazil based on WHO CHOICE’s bed-day costs. This reported cost was 4.899 times as high as the upper bound ($230) of the WHO CHOICE estimates for Brazil. Because we suspected the estimates for Bangladesh and Nigeria may also be low, we created alternative estimates of hospital costs by multiplying the upper bounds of the WHO CHOICE cost estimates for those countries by 4.899, yielding $257 for Bangladesh and $1,164 for Nigeria. We used the ratio of Brazil’s reported cost to its standard error, 6.0628, to estimate standard errors for the alternative estimates for Bangladesh and Nigeria.

References:

1. Colin Sanderson, of the London School of Hygiene and Tropical Medicine, provided us with vaccination probabilities by week of age modeled from Demographic and Health Survey data for Bangladesh (2011 DHS) and Nigeria (2008 DHS); the modeling is similar to that in [Ref 2] but the data are more recent
2. [Clark A](http://www.ncbi.nlm.nih.gov/pubmed/?term=Clark A%5BAuthor%5D&cauthor=true&cauthor_uid=19303633), [Sanderson C](http://www.ncbi.nlm.nih.gov/pubmed/?term=Sanderson C%5BAuthor%5D&cauthor=true&cauthor_uid=19303633). Timing of children's vaccinations in 45 low-income and middle-income countries: an analysis of survey data. [*Lancet*.](http://www.ncbi.nlm.nih.gov/pubmed/?term=The+Lancet+(2009+May+2%3B373(9674)%3A1543-9)) 2009;373(9674):1543-9
3. GOIANIA. Sistema de Controle do Atendimento Ambulatorial (SICAA). Pertussis doses by age, children < 1 year, 2014. Unpublished data, made available by the Goiânia Municipal Health Department.
4. BRASIL. DATASUS. Programa Nacional de Imunizações. Coberturas Nacionais de Vacinação. 2014. Available online at: http://tabnet.datasus.gov.br/cgi/deftohtm.exe? pni/cnv/cpniuf.def
5. National Institute of Population Research and Training (NIPORT), Mitra and Associates, and ICF International.2016*. Bangladesh Demographic and Health Survey 2014*. Dhaka, Bangladesh, and Rockville, Maryland, USA: NIPORT, Mitra and Associates, and ICF International.
6. National Population Commission (NPC) [Nigeria] and ICF International. 2014. *Nigeria Demographic and Health Survey 2013.* Abuja, Nigeria, and Rockville, Maryland, USA: NPC and ICF International.
7. World Health Organization (WHO). Immunization Coverage. Available at: <http://www.who.int/immunization/monitoring_surveillance/data/en/>. Last Accessed: March, 2016
8. Brasil. Programa Nacional de Imunizações (PNI). Immunization coverage. Td/TdaP vaccine in pregnant women, 2014. Unpublished data, made available by the Surveillance Secretariat, Brazilian Ministry of Health.
9. Amirthalingam G, Andrews N, Campbell H et al. Effectiveness of maternal pertussis vaccination in England: An observational study. *Lancet* 2014; 384: 1521-28.
10. Juretzko P, von Kries R, Hermann M, et al. Effectiveness of acellular pertussis vaccine assessed by hospital-based active surveillance in Germany. *Clin Infect Dis* 2002;35:162-7.
11. United Nations, Department of Economic and Social Affairs, Population Division (2015). World Population Prospects: The 2015 Revision, DVD Edition. File MORT/16-1: Life expectancy at exact age, e(x), for both sexes combined, by major area, region and country, 1950-2100. Available at: https://esa.un.org/unpd/wpp/Download/Standard/Population/. Last Accessed: February 12, 2015
12. United Nations, Department of Economic and Social Affairs, Population Division (2015). World Population Prospects: The 2015 Revision, DVD Edition*. File MORT/15-1: Life table survivors at exact age, l(x), for both sexes combined, by major area, region and country, 1950-2100.* Available at: <https://esa.un.org/unpd/wpp/Download/Standard/Population/>. Last Accessed: February , 2015
13. United Nations Inter-agency Group for Child Mortality Estimation (UN IGCME). Child Mortality Estimates. Available at: <http://www.childmortality.org/>. Last Accessed: October , 2015
14. Brasil. DATASUS. Sistema de Informação de Hospitalizações (SIH-SUS). SUS Hospital Morbidity. CID-10 Pertussis. Hospitalizations by year service, by place of residence, Brazil, 2014; <http://tabnet.datasus.gov.br/cgi/tabcgi.exe?sih/cnv/nruf.def>; 2014. Last accessed February 2016
15. World Health Organization (WHO). Choosing Interventions that are Cost Effective (WHO-CHOICE). Country-specific Unit Costs. Available at: http://www.who.int/choice/country/country_specific/en/. Last Accessed on: September, 2015
16. World Health Organization (WHO). The cMYP Immunization Financing Database. Available at: <http://www.who.int/immunization/programmes_systems/financing/countries/en/> Last Accessed: September, 2015.
17. [Janusz CB](http://www.ncbi.nlm.nih.gov/pubmed/?term=Janusz CB%5BAuthor%5D&cauthor=true&cauthor_uid=25919175), [Castañeda-Orjuela C](http://www.ncbi.nlm.nih.gov/pubmed/?term=Castañeda-Orjuela C%5BAuthor%5D&cauthor=true&cauthor_uid=25919175), [Molina Aguilera IB](http://www.ncbi.nlm.nih.gov/pubmed/?term=Molina Aguilera IB%5BAuthor%5D&cauthor=true&cauthor_uid=25919175) et al. Examining the cost of delivering routine immunization in Honduras. [*Vaccine*.](http://www.ncbi.nlm.nih.gov/pubmed/25919175) 2015;33 Suppl 1:A53-9
18. Castañeda-Orjuela C, Romero M, Arce P et al. Using standardized tools to improve immunization costing data for program planning: the cost of the Colombian Expanded Program on Immunization. Vaccine. 2013;31 Suppl 3:C72-9.
19. UNICEF. Supplies and Logistics. Vaccine Price Data. Available at: http://www.unicef.org/supply/index_57476.html. Last Accessed: November, 2015.
20. UNICEF. Supplies and Logistics. Auto-Disable (AD) and Re-Use Prevention (RUP) Syringes and Safety Boxes - current price data. Available at: http://www.unicef.org/supply/index_62309.html. Last Accessed: March, 2016.
21. UNICEF. Supplies and Logistics. Procurement Services. Available at: http://www.unicef.org/supply/index_procurement_services.html. Last Accessed: March, 2016
22. Maternal and infant vaccine prices for Brazil from the Pan American Health Organization Revolving Fund 2015 price list [http://www.**paho**.org](http://www.paho.org/). Last accessed December 2015.
23. World Health Organization (WHO). [Immunization schedules by antigens - World Health Organization](http://apps.who.int/immunization_monitoring/globalsummary/schedules). Available at: <http://apps.who.int/immunization_monitoring/globalsummary/schedules>. Last Accessed January , 2016
24. World Bank (WB) national accounts data, and OECD National Accounts data files. World Development Indicators. *Inflation, GDP deflator (annual %). Last Updated12/22/2015.* Available at: http://data.worldbank.org/indicator/NY.GDP.DEFL.KD.ZG. Last Accessed March, 2016
25. International Monetary Fund, International Financial Statistics. World Development Indicators. *Official exchange rate (LCU per US$, period average). Last Updated12/22/2015.* Available at: <http://data.worldbank.org/indicator/PA.NUS.FCRF>. Last Accessed: March, 2016
26. Sinha A, Kim S, Ginsberg G et al. Economic burden of acute lower respiratory tract infection in South African children. Paediatr Int Child Health. 2012;32(2):65-73
27. From our unpublished systematic review of infant pertussis incidence and mortality in LMICs.
